# Late-onset thymidine kinase 2 deficiency: a review of 18 cases

**DOI:** 10.1186/s13023-019-1071-z

**Published:** 2019-05-06

**Authors:** Cristina Domínguez-González, Aurelio Hernández-Laín, Eloy Rivas, Ana Hernández-Voth, Javier Sayas Catalán, Roberto Fernández-Torrón, Carmen Fuiza-Luces, Jorge García García, Germán Morís, Montse Olivé, Frances Miralles, Jordi Díaz-Manera, Candela Caballero, Bosco Méndez-Ferrer, Ramon Martí, Elena García Arumi, María Carmen Badosa, Jesús Esteban, Cecilia Jimenez-Mallebrera, Alberto Blazquez Encinar, Joaquín Arenas, Michio Hirano, Miguel Ángel Martin, Carmen Paradas

**Affiliations:** 1 Neurology department, Neuromuscular disorders Unit, 12 de Octubre Hospital, Madrid, Spain; 2 Research Institute i+12, 12 de Octubre Hospital, Madrid, Spain; 3 Biomedical Network Research Centre on Rare Diseases (CIBERER), Instituto de Salud Carlos III, Madrid, Spain; 4 Neuropathology, Pathology Department, Neuromuscular disorders Unit, 12 de Octubre Hospital, Madrid, Spain; 5 Pathological Anatomic Department, Neuromuscular Disorders Unit, Instituto de Biomedicina de Sevilla, Hospital U. Virgen del Rocío, CSIC, Universidad de Sevilla, Sevilla, Spain; 6 Neumology department, Neuromuscular disorders Unit, 12 de Octubre Hospital, Madrid, Spain; 7 Neurology Department, Neuromuscular disorders Unit, Hospital de Donostia, San Sebastian, Spain; 8 Mitochondrial and Neuromuscular Diseases Laboratory, Research Institute of Hospital ‘12 de Octubre’ (‘i+12’), Madrid, Spain; 9 Neurology Department, Hospital de Albacete, Albacete, Spain; 10 Neurology Department, Neuromuscular disorders Unit, Hospital Central de Asturias, Oviedo, Spain; 11 Pathological Anatomy Department, Neuromuscular disorders unit, IDIBELL-Hospital de Bellvitge, Barcelona, Spain; 12 Neurology department, Neuromuscular disorders unit, Hospital Universitari Son Espases, Palma, Spain; 13 Neurology department, Neuromuscular disorders unit, Hospital de la Santa Creu I Sant Pau, Barcelona, Spain; 14 Respiratory Department, Instituto de Biomedicina de Sevilla, Hospital U. Virgen del Rocío, CSIC, CIBERES, Universidad de Sevilla, Sevilla, Spain; 15 Rehabilitation Department, Hospital Virgen del Rocio, Sevilla, Spain; 16 Research group on Neuromuscular and Mitochondrial Diseases, Valld’Hebron Research Institute, Universitat Autònoma de Barcelona, Barcelona, Spain; 17 Neuromuscular Unit, Neurology Department, Institut de Recerca Sant Joan de Déu, Hospital Sant Joan de Déu, Barcelona, Spain; 18 Department of Neurology, H. Houston Merritt Center, Columbia University Medical Center, New York, New York, USA; 19 Neurology Department, Neuromuscular Disorders Unit, Instituto de Biomedicina de Sevilla, Hospital U. Virgen del Rocío, CSIC, Universidad de Sevilla, Avd. Manuel Siurot s/n, 41013 Sevilla, Spain; 20 Biomedical Network Research Centre on Neurodegenerative Diseases (CIBERNED), Madrid, Spain

**Keywords:** TK2 deficiency, Mitochondrial myopathy, Multiple deletions

## Abstract

**Background:**

TK2 gene encodes for mitochondrial thymidine kinase, which phosphorylates the pyrimidine nucleosides thymidine and deoxycytidine. Recessive mutations in the TK2 gene are responsible for the ‘myopathic form’ of the mitochondrial depletion/multiple deletions syndrome, with a wide spectrum of severity.

**Methods:**

We describe 18 patients with mitochondrial myopathy due to mutations in the TK2 gene with absence of clinical symptoms until the age of 12.

**Results:**

The mean age of onset was 31 years. The first symptom was muscle limb weakness in 10/18, eyelid ptosis in 6/18, and respiratory insufficiency in 2/18. All patients developed variable muscle weakness during the evolution of the disease. Half of patients presented difficulty in swallowing. All patients showed evidence of respiratory muscle weakness, with need for non-invasive Mechanical Ventilation in 12/18. Four patients had deceased, all of them due to respiratory insufficiency. We identified common radiological features in muscle magnetic resonance, where the most severely affected muscles were the gluteus maximus, semitendinosus and sartorius. On muscle biopsies typical signs of mitochondrial dysfunction were associated with dystrophic changes. All mutations identified were previously reported, being the most frequent the in-frame deletion p.Lys202del. All cases showed multiple mtDNA deletions but mtDNA depletion was present only in two patients.

**Conclusions:**

The late-onset is the less frequent form of presentation of the TK2 deficiency and its natural history is not well known. Patients with late onset TK2 deficiency have a consistent and recognizable clinical phenotype and a poor prognosis, due to the high risk of early and progressive respiratory insufficiency.

## Background

Defects in the maintenance and repair of mitochondrial DNA (mtDNA) result in an emerging and heterogeneous group of mitochondrial disorders, caused by alterations of the nuclear genes involved in mtDNA replication [1–3]. This group includes defects in enzymes involved in the maintenance of the balanced pool of deoxynucleotides of the mitochondria, which are crucial in the biosynthesis of the mitochondrial genome and have therapeutic implications [4, 5]. The disrupted synthesis of mtDNA results in qualitative (multiple deletions) and/or quantitative (a drastic decrease in the number of copies or depletion) defects of the mtDNA. In particular, one of the ‘myopathic forms’ of the mitochondrial depletion/multiple deletions syndromes is caused by mutations in the *TK2* gene which encodes for mitochondrial thymidine kinase, which phosphorylates the pyrimidine nucleosides thymidine (dT) and deoxycytidine (dC) [1, 6].

Recessive mutations in the *TK2* gene (MIM# 609560) are responsible for diverse clinical presentations mainly characterized by progressive muscle weakness, dysphagia and respiratory involvement with a wide spectrum severity and of age of onset. TK2 deficiency was initially described by Saada, et al. in 2001 [6] in four children with a severe myopathy associated with depletion of the mtDNA. Since then, a number of cases have been reported depicting a heterogeneous clinical presentation with a continuum spectrum of the disease, which includes early-onset extremely severe and rapidly progressive forms with survival of less than two years, to less severe forms with late or very late onset, and a variably slower rate of progression [7, 8]. In 2012, Tyynismaa, et al. reported the first two cases with mutations in the *TK2* gene with onset in the fifth decade of life, manifesting chronic progressive external ophthalmoplegia (CPEO) associated with limb muscle weakness and dysphagia [9]. A recent publication that included 92 patients describing the natural history of this disorder proposed the classification of three clinical forms according to ages-at-onset: infantile (< 1 year-old), childhood (1–12) and late (> 12 years) onset. Nearly 40% of the reported TK2 cases presented with the symptoms prior to the age of 1, in another 41% the onset occurred between the ages of one and 12, and only in 19% of patients did the symptoms appeared after the age of 12 [7]. A subsequent retrospective review, with similar frequencies for those three subgroups, included eleven new cases of which only three were classified as late-onset [8]. So far, the natural history of patients with late onset TK2 deficiency has not been defined in detail.

Here, we report on the clinical features and assessments in a large series of 18 patients with late-onset TK2 deficiency, the less known and poorest defined form of this disease, to further characterize this patient subgroup. Expanding the natural history and prognosis of late-onset TK2 deficiency will facilitate earlier diagnosis and identification for treatment with therapies under clinical development.

## Methods

### Patients

We describe the phenotypic features of 16 Spanish and 2 US patients with mitochondrial myopathy due to mutations in the *TK2* gene with the absence of clinical symptoms until the age of 12. The series include three pairs of siblings (P3-P4, P6-P10 and P14-P15). Partial data from five patients have been previously published elsewhere (P1, P5, P9 [7], P3 and P12 [10]).

### Clinical evaluation

The electronic records were reviewed to collect information about the age of onset, initial symptoms, severity, distribution and progression of the muscle weakness and extra-muscular symptoms. We gathered information from the latest neurological examination registered including, when available, the Muscle Research Council (MRC) scale to assess the muscle strength and the 6 min walk test (6MWT) for functional evaluation.

### Respiratory assessment

The latest value of the forced vital capacity (FVC) in seated and supine position, maximum inspiratory pressure (MIP), blood gas analysis, nocturnal ventilation (assessed with nocturnal pulse oximetry and/or capnography [11] and the need for mechanical ventilation (MV) type and hours of use were recorded.

### Laboratory tests

CK (creatine kinase) and lactate levels were quantified in serum in basal conditions, at diagnosis. GDF-15 (growth/differentiation factor-15) levels were quantified in plasma samples using human GDF-15 quantitative ELISA kit (R&D Biosystems) according to the manufacturer’s instructions.

### Muscle MRI

Muscle MRI was performed in 8 of the 18 patients. All of them were scanned in a 1.5 T MR scanner (Siemens). Lower limb axial T1-weighted sequences were used for morphological analysis and short-tau inversion recovery (STIR) sequences were examined to detect muscle edema. The muscle MRI studies were evaluated by the same neurologist (R F-T) with wide experience in neuromuscular disorders. The evaluator was blind regarding the clinical manifestations. He scored pelvic, thigh and lower leg muscles in axial T1-sequences with the semiquantitative Mercuri visual scale (MVS) modified by Fisher [12]: 0: Normal appearance; 1: Mild involvement, less than 30% of individual muscle volume; 2: Moderate involvement, 30–60% of individual muscle volumes; 3: Severe involvement, > 60% of individual muscle; 4: End stage, all the muscle is severely affected, replaced by increased density of connective tissue and fat, with only a rim of fascia and neurovascular structures distinguishable. We compared median value of muscle fatty replacement using the Wilcoxon-Mann-Whitney test. Statistical analyses were performed using IBM SPSS Statistics, V.22 (IBM, Armonk, New York, USA).

### Aerobic exercise testing

Exercise testing was performed in 5 patients on a cycle ergometer, following a ramp-like protocol (workload increases of 1 W every 6 s [averaging 10 W·min^− 1^] starting from an initial load of 0 W, with a pedal cadence of 60–70 rpm throughout the test). Gas-exchange variables were collected breath-by-breath with an automated metabolic cart (Quark CPET, COSMED, Rome, Italy). The peak oxygen uptake (VO_2_ peak) was computed as the highest value obtained for any 10-s period during the tests [13].

### Muscle biopsy

Muscle samples were obtained by open biopsy and processed following the standard procedures: Hematoxylin and eosin (H&E), modified Gomori trichrome, ATPase (adenosine triphosphatase), NADH (nicotinamide adenine dehydrogenase), SDH (succinate dehydrogenase), COX (cytochrome C oxidase), and COX-SDH stains were performed in all available samples. Respiratory chain enzyme activity levels were recorded when available.

### Genetic studies

Molecular diagnosis was performed either by direct Sanger sequencing of exons and intron/exon boundaries of the *TK2* gene, or by customized next generation sequencing (NGS) panels. Patient’s skeletal muscle mtDNA deletions were investigated by long-range PCR (polymerase chain reaction) and/or Southern blot, and mtDNA copy number was assessed by quantitative PCR as previously described [10, 14].

The study was approved by the institutional review board of every centre and all patients signed an informed consent for the anonymous publication of this data.

## Results

### Clinical manifestations (Table 1)

We included 18 patients (6 male, 12 female). The mean age-at-onset was 31 years (range 12 to 60 years) with a mean age at diagnosis of 48.5 years (range 23 to 73 years) resulting in an average of 17.4 years between the onset of the disease until reaching a genetic diagnosis (range 1 to 44 years). The mean duration of the disease was 19.8 years (range 6 to 44 years). Four patients from the series were deceased, all of them due to respiratory insufficiency a mean of two decades after the onset.

**Table 1:** Clinical manifestations summary

ID	Age At Onset	Gender	Current Age	Clinical Assessments											Respiratory Assessments			Other manifestations
				Ptosis	CPEO	Facial Weakness	Dysphagia	Neck flexor weakness	Limb Weakness	Wheelchair-bound	6MWT (Meters)	PEG (AT AGE)	Orthopnea	BMI	Sitting FVC (%)	FVC Sitting/Supine (%)	Hours on MV (AT AGE)	
1	12	F	32	+	–	+	Yes	+++	+	No	530	No	No	13.86	46	−8	8 (32)	–
2	20	F	33	–	–	+	Yes	+++	+	No	390	Yes (32)	Yes	17.9	26	−22	8–9 (28)	Hepatopathy
3	30	F	61	+	–	+	Yes	++	+	No	386	No	Yes	27.12	71	−14	8 (61)	PNP-S
4	60	F	73	+	+	++	Yes	++	+	No	345	No	Yes	26	69	NA	8 (73)	Hearing loss PNP-S
5	50	M	50	++	–	+	No	+	+	No	475	No	Yes	27.6	47	NA	12 (50)	PNP-S
6	14	F	58	++	++	++	No	+++	+	No	450	No	Yes	24.5	51	−28	8 (56)	–
7	40	M	Death at 68	++	++	+	Yes	+++	++	Yes	NA	Yes (68)	Yes	23.7	48	NA	10–12 (49)	Hearing loss PNP-S Vocal cord palsy
8	50	F	63	+	+	++	No	+	+	No	417	No	Yes	28.7	103	−3	–	PNP-S
9	23	F	Death at 40	–	–	+	Yes	+++	++	Yes	NA	Yes (39)	Yes	15	28	NA	22 (30)	–
10	14	M	Death at 49	+	–	–	Yes	++	+++	Yes	NA	Yes (42)	Yes	17	32	NA	24 (42)	–
11	40	M	51	+	–	+	No	–	+	No	600	No	No	26.3	70	−10	–	–
12	30	M	60	++	–	+	Yes	NA	+	No	NA	No	No	23	75	NA	–	Vocal cord palsy
13	48	F	Death at 67	+	+	+	No	+++	+++	Yes	NA	No	Yes	NA	43	NA	12 (58)	–
14	15	M	26	+	+	+	Yes	+	+	No	413	No	No	NA	72	−10	–	Hearing loss PNP-S
15	12	F	31	+	+	+	No	+	+	No	428	No	No	NA	75	−12	–	PNP-S
16	30	F	43	++	+	+	No	+	+	No	425	No	Yes	NA	69	2	8 (43)	–
17	45	F	51	++	+	++	Yes	+	+	No	NA	No	No	NA	NA	NA	–	NA
18	25	F	58	++	++	++	Yes	++	++	No	228	Yes (39)	Yes	NA	17	NA	11 (59)	NA

*NA* Not available. Ptosis: ++ History of eyelid surgery, + mild, − no ptosis. *CPEO* chronic progressive external ophthalmoplegia: ++ complete, + partial, − no ocular weakness. Facial weakness: ++ severe, + mild, − no facial weakness. Neck flexor weakness; +++ unable to lift head in supine position, ++ 3 on Medical Research Council (MRC) scale, + 4 on MRC scale, − no neck flexor weakness. Limb weakness: +++ 1–2 on MRC scale, ++ 3 on MRC scale, + 4 on MRC scale, − no weakness. *6MWT* six-minute walking test, *FVC* forced vital capacity, *MV* Mechanical ventilation, *PNP-S* sensory polyneuropathy, *BMI* body mass index

The first symptom was muscle limb weakness in 10/18 (55.6%), eyelid ptosis in 6/18 (33%) (two patients also presented ophthalmoparesis), and respiratory insufficiency in 2/18 (11.1%). All patients developed muscle weakness during the evolution of the disease, 17/18 showing proximal and distal limb muscle weakness, 1/18 with only distal limb weakness, and 16/18 axial involvement. It is noteworthy that neck flexor weakness was clearly more severe than limb weakness (mean, 2.14 on the MRC scale).

The following muscle groups were the most frequently affected, in a symmetrical manner: shoulder abductor (mean, 4 on the MRC scale), hip flexor (mean, 3.75 on the MRC scale) and hip extensor (mean, 3.87 for both on the MRC scale) and finger extensor muscles (mean, 4.14 on the MRC scale). Four patients (22%) lost the ability to walk without support. Facial musculature was symmetrically affected in 17 patients (94.4%), with predominance of the orbicular oculis muscle. 16/18 of the patients (88.9%) also had symmetrical eyelid ptosis of variable severity, with this being the first symptom in 6 patients (33.3%). Six of them required surgical blepharoplasty due to vision impairment. Nine patients had CPEO.

The majority (11/18) had difficulty in swallowing, which resulted in severe weight loss and/or detriment to the safety of oral feeding in 6 cases, requiring percutaneous gastrostomy tube in 5 cases (27.8%) on average 19.6 years after the onset of the disease (ranging from 12 to 28 years).

Other clinical manifestations included sensory axonal polyneuropathy (7/18;38.9%), neurosensory hearing loss (3/18;16.6%) and dysphonia due to vocal cord palsy (2/18;11.1%). No patient had cardiomyopathy.

### Respiratory function

FVC at diagnosis from the total cohort was 55.4% (ranging from 17 to 103) with a mean decrease of FVC in supine position of 8% (ranging from 0 to 14), and a mean MIP of 36.8% (ranging from 20 to 101%), independent of the associated muscle symptoms. From a respiratory perspective, the high frequency of complications should be noted, with need for non-invasive MV in 12/18 patients (66.6%). The mean use of the MV was 11.6 h per day (ranging from 8 to 24 h). Eight out of the 12 patients with MV (66.6%) presented with acute respiratory insufficiency following a routine upper respiratory infection as the first manifestation of the disease. None of these cases had any prior respiratory symptoms; however, once detected, they required MV due to hypercapnia secondary to alveolar hypoventilation. Although limb muscle weakness and/or eyelid ptosis were already present at the onset of respiratory insufficiency, those neuromuscular symptoms had not prompted a neurology consultation in any of the eight patients. Thus, the respiratory involvement resulted in the diagnosis of an underlying myopathy in these patients; the mean FVC was of 40.8% (range from 28 to 58) at the time of diagnosis. Of the six patients who did not needed MV, all showed evidence of respiratory muscle weakness on functional tests, although only one of them (P8) reported respiratory symptoms (orthopnoea), suggesting diaphragmatic weakness. This patient displayed ptosis and CPEO at the age of 50 associated with moderate axial and proximal limb muscle weakness (4 on the MRC scale). Strikingly, although the functional respiratory tests and nocturnal pulse oximetry were normal (FVC seated 103%, FVC decubitus 100% and MIP 101%) nocturnal transcutaneous capnography revealed high mean levels of carbon dioxide (CO_2_, mean of 48 mmHg, with a maximum peak of 54 mmHg).

Four patients died of respiratory insufficiency at mean age of 56 years (ranging from 40 to 68), and a mean of 24 years after the onset of their initial symptoms (ranging from 17 to 35).

### CK and lactate levels (Table 2)

94.4% of patients had increased variable serum CK levels ranging from 190 to 2435 UI/l (normal levels < 170 UI/l)), and 16.7% showed levels 10-fold above the upper normal limit. Serum lactate levels were measured in basal conditions in 12 of the 18 cases. Of these, only three (25%) displayed slightly increased levels (1.4-2x above the upper normal limit).

**Table 2:** Biochemical and molecular characteristics

ID	Mutation		Muscle Biopsy	Multiple Deletions	Residual mtDNA (%)	Respiratory Chain Enzyme Activity	CK (UI/l)	GDF-15 (pg/mL)	Lactate (mmol/l)
	Allele 1	Allele 2							
1	c.323C > T (p.Thr108Met)	c.323C > T (p.Thr108Met)	Yes	Yes	17	CI, CIII and CIV deficit	2435	2423	1.95
2	c.323C > T (p.Thr108Met)	c.323C > T (p.Thr108Met)	Yes	Yes	39	Normal	303	2439	2.3
3	c.604–606 AAGdel (p.Lys202del)	c.604–606 AAGdel (p.Lys202del)	Yes	Yes	60	CIII deficit	294	1695	2.6
4	c.604–606 AAGdel (p.Lys202del)	c.604–606 AAGdel (p.Lys202del)	ND	ND	NA	ND	647	2483	2.2
5	c.604–606 AAGdel (p.Lys202del)	c.604–606 AAGdel (p.Lys202del)	Yes	Yes	66	Normal	357	1529	1.5
6	c.323C > T (p.Thr108Met)	c.323C > T (p.Thr108Met)	Yes	Yes	19	Normal	425	NA	1.6
7	c.604–606 AAGdel (p.Lys202del)	c.604–606 AAGdel (p.Lys202del)	Yes	Yes	33	NA	568	NA	2.6
8	c.604–606 AAGdel (p.Lys202del)	c.604–606 AAGdel (p.Lys202del)	Yes	Yes	NA	NA	405	NA	2.4
9	c.323C > T (p.Thr108Met)	c.323C > T (p.Thr108Met)	Yes	Yes	35	CI, CIII and CIV deficit	190	NA	NA
10	c.323C > T (p.Thr108Met)	c.323C > T (p.Thr108Met)	Yes	NA	NA	Normal	405	NA	3
11	c.604–606 AAGdel (p.Lys202del)	c.604–606 AAGdel (p.Lys202del)	Yes	Yes	NA	ND	266	NA	NA
12	c.604–606 AAGdel (p.Lys202del)	c.604–606 AAGdel (p.Lys202del)	Yes	Yes	NA	ND	350	NA	NA
13	c.388C > T (p.Arg130Trp)	c.415G > A (p.Ala139Thr)	Yes	Yes	NA	CI, CIII and CIV deficit	170	NA	4.14
14	c.623A > G (p.Tyr208Cys)	c.623A > G (p.Tyr208Cys)	Yes	Yes	NA	CI, CIII and CIV deficit	1739	NA	NA
15	c.623A > G (p.Tyr208Cys)	c.623A > G (p.Tyr208Cys)	ND	NA	NA	ND	381	NA	NA
16	c.323C > T (p.Thr108Met)	c.323C > T (p.Thr108Met)	Yes	Yes	53	Normal	233	NA	1.77
17	c.469_470insTGGG (p.Asp157Valfs*11)	c.156 + 6 T > G	Yes	Yes	50	NA	537	NA	NA
18	c.604–606 AAGdel (p.Lys202del)	c.604–606 AAGdel (p.Lys202del)	NA	NA	NA	NA	1348	NA	NA

*NA* not available, *ND* not done, *CK* creatine kinase, *GDF-15* Growth differentiation factor-15

### GDF-15 levels

GDF-15, a biomarker identified in the analysis of transcriptomic profiling of TK2 deficient human skeletal muscle [15], has been proven useful in the diagnosis of mitochondrial myopathies [16], being especially increased in patients with mitochondrial TK2 deficiency [17]. Serum levels of GDF-15 were increased in 5 out of 5 cases analysed (100%), ranging from 1529 to 2438 pg/mL (2113 pg/mL ± 462, mean ± standard deviation, upper limit of normal =550 pg/mL) [16].

### Muscle MRI findings

**Fig. 1:**
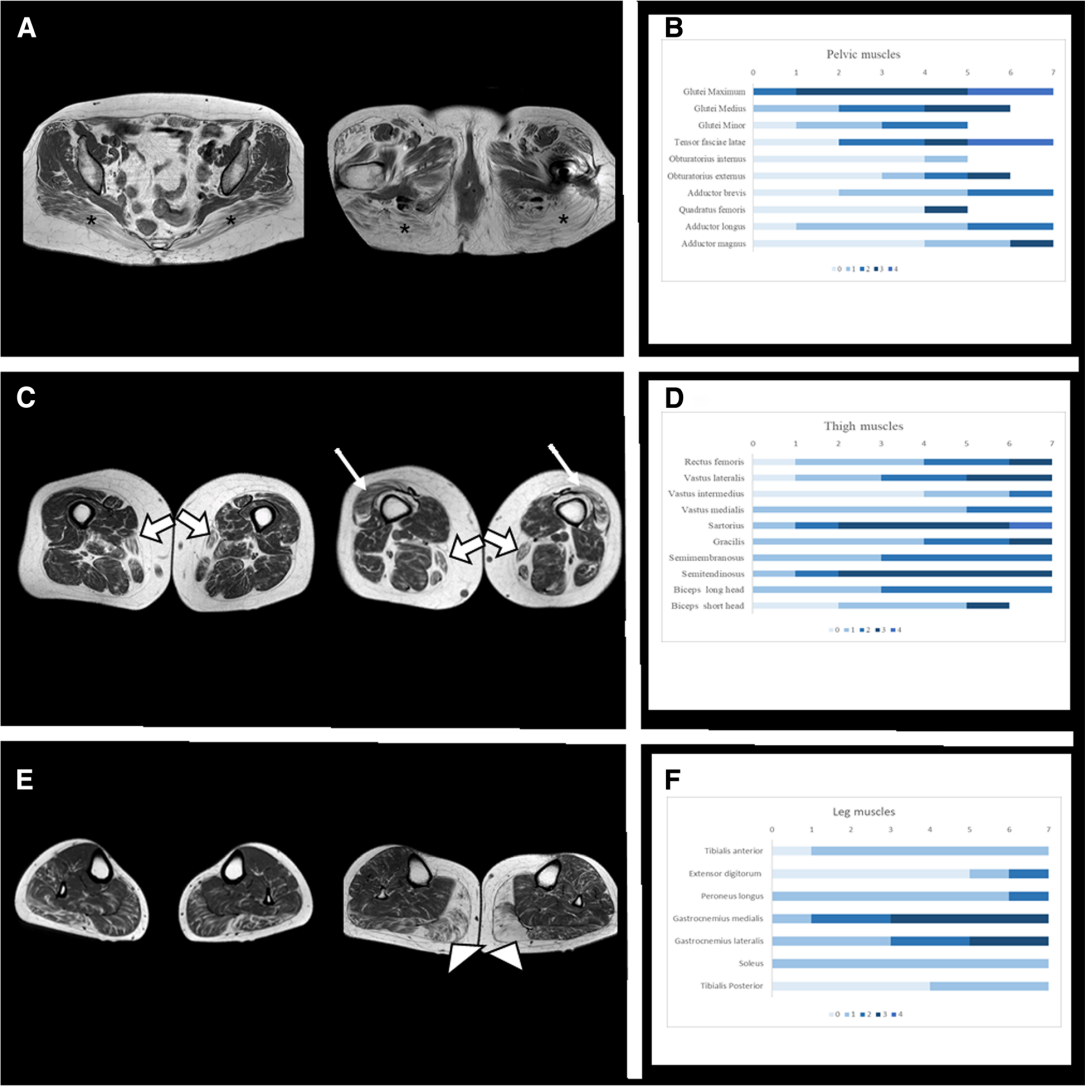
Axial T1 muscle MRI and bar charts with Mercuri Visual Scale (MVS) distribution for 7 patients and per anatomical region. **a**, Axial T1 muscle MRI in pelvis: These two consecutive slices from different patients are showing that the gluteus maximus (marked with asterisk) is the most affected muscle. Tensor fascia latae is affected while obturator and quatratus femoris are less affected. **b**, Bar chart MVS fat replacement in pelvis: MVS (0: no fat replacement, 4: the muscle is completely replaced) for all patients. Gluteus maximus is the most affected muscle, followed by tensor fascia latae. **c**, Axial T1 muscle MRI in thighs: These two slices from two different patients are showing the fat replacement of sartorius (wide white arrow) and vastus lateralis (thin white arrow). Other muscles like semitendinosus, semimembranosus and gracilis are also moderately affected. **d**, Bar chart MVS fat replacement in thighs: MVS for all patients. Sartorius, semimembranosus, semitendinosus, gracilis and vastus lateralis are the most affected muscles. Sartorius and gracilis are affected in all patients. **e**, Axial T1 muscle MRI in legs: These two slices from two different patients are showing the fat replacement of gastrocnemius medialis (white arrow head). Gastrocnemius lateralis and soleus are also moderately affected. Tibialis anterior and tibialis posterior are the least affected. **f**, Bar chart MVS fat replacement in legs: MVS for all patients. Gastrocnemius medialis and lateralis are the most affected muscles in legs. Tibialis anterior, extensoris digitorum and tibialis posterior are the least affected muscles

It was performed in 8 patients. Mean age at muscle MRI was 46.4 years old (range: 23–73). Mean disease duration at the time of the scan was 18 years (range 10–31). The most severely affected muscles in axial T1-weighted sequences were the gluteus maximus, semitendinosus, sartorius and gastrocnemius medialis (median MVS: 3). Of these, only the gluteus maximus and sartorius were affected in all patients. Apart from the later, gluteus medius, adductor magnus and semitendinosus were also moderately affected in the thighs and gastrocnemius lateralis in the legs (median MVS: 2). No muscle fat infiltration was observed in obturator, quadratus femoris, extensoris digitorum and tibialis posterior (Fig. 1). The fat replacement followed a diffuse pattern and no focal areas of fat infiltration were detected. We did not observe statistical differences regarding asymmetric involvement. STIR sequence was normal in all patients.

### Aerobic exercise testing

In addition to weakness, one of the most frequent clinical manifestations in the mitochondrial myopathies is poor exercise capacity [18].The latter is reflected by low levels of VO_2_peak or by poor muscle-oxygen extraction (as assessed with near-infrared spectroscopy) during graded cycle-ergometer/treadmill testing [19]. Aerobic exercise testing was performed on a cycle ergometer in five patients. The mean ± SD VO_2_ peak obtained was 14.8 ± 3.2 mL/kg^− 1^/min^− 1^, with normal consumption values of 40.0 ± 9.5 mL/kg^− 1^/min^− 1^ [20].

### Muscle biopsies

**Fig. 2:**
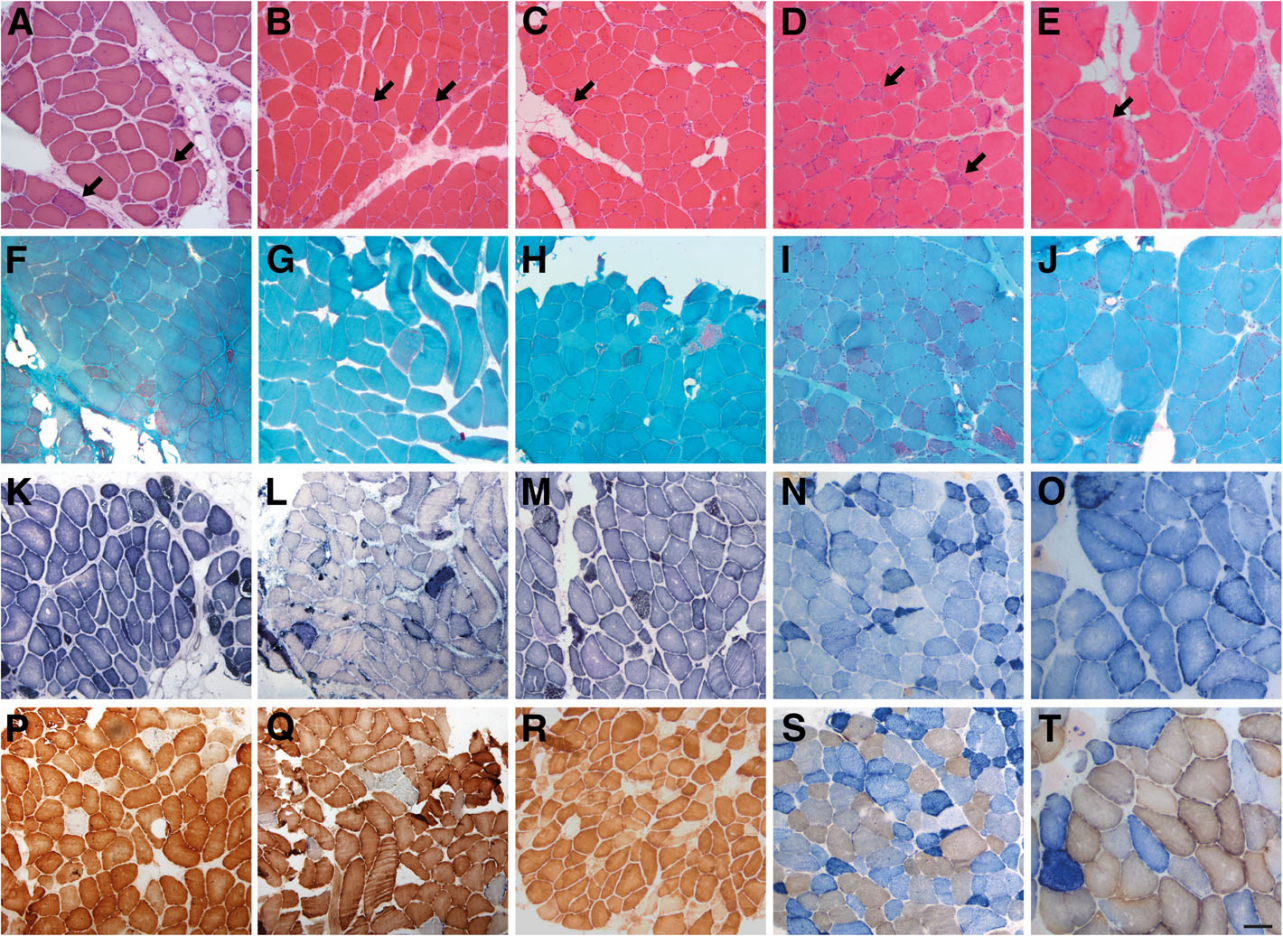
Morphological alterations in muscle biopsies from patients P1 (**a**, **f**, **k**, **p**), P5 (**b**, **g**, **l**, **q**), P9 (**c**, **h**, **m**, **r**), P14 (D, **i**, **n**, **s**) and P16 (**e**, **j**, **o**, **t**). **a-e** H&E shows dystrophic features in all cases with mild endomysal fibrosis, adipose tissue replacement, atrophy and necrotic fibers. Ragged-red fibers are frequently identified in all muscle samples (arrows). **f-j** Gomori trichrome showed the characteristic ragged-red fibers in all the biopsies. **k-o** Succinate dehydrogenase (SDH) reveals an increase of the oxidative staining in numerous fibers. **p-t** Frequent cytochrome C oxidase (COX) deficient fibers are present in variable proportion in the different cases (**p** and **r**, COX staining; **o**, **s** and **t**, COX-SDH combined staining). Scale bar =100 μm

Muscle biopsies were performed in 16 patients, 11 were available to re-analysis. The morphological study revealed numerous ragged-red fibers in 100% of the biopsies, which were hyper-reactive with SDH reaction and usually COX-deficient. COX-deficient fibers accounted for approximately 5–15% of all fibers. Frequently these muscles also showed dystrophic features with frequent necrotic fibers, some with phagocytosis, and increased endomysial connective tissue (present in 7 out of 11 biopsies revised). Marked type I fiber predominance was also observed in 2 patients (Fig. 2). These findings differ from the usual pattern displayed in other mitochondrial myopathies, where the typical signs of mitochondrial proliferation and dysfunction are not associated with other relevant changes in muscle histology structure [21]. We have results of the analysis of the enzymatic activity of respiratory chain complexes of 10 patients. Only in half of them a reduction in the activity of one or more enzymatic complexes were identified (Table 2).

### Genetic studies

All patients harbored biallelic mutations in the *TK2* gene (Ref.Seq. NM_004614.4) (Table 2). Most patients (16/18;88.9%) were homozygous. All mutations were previously reported [7, 8], with the in-frame deletion p.Lys202del (c.604_606AAGdel) being the most frequent (16/36 alleles; 44.4%), followed by the missense mutation p.Thr108Met (c.323C > T) (12/36;27.8%). Additionally, three missense mutations were identified in 3 patients: p.Arg130Trp (c.388C > T), p.Ala139Thr (c.415G > A), and p.Tyr208Cys (c.623A > G). Finally, one patient harbored a frameshift mutation p.Asp157Valfs*11(c.469-470insTGGG) in compound heterozygosis with a splice site mutation c.156 + 6 T > G. Genetic data from patients P1, P2, P5, P9 and P12, were previously reported [7, 10]. Muscle mtDNA copy-number was studied in 9 patients and severe mtDNA depletion was detected in only two (17% of residual mtDNA in P1 and 19% of residual mtDNA in P6). Fourteen out of 14 patients (100%) showed the presence of multiple mtDNA deletions in muscle.

## Discussion

The late-onset presentation of TK2 deficiency is the least frequent clinical mode of presentation known. These patients are considered to have a milder presentation than those with infancy and childhood onset disease, however, few cases have been described to date and those reported were not extensively explored. So far, 17 patients with late-onset were reported to harbour *TK2* biallelic mutations [7–10, 22]. However clinical details were scarce, heterogeneous, and reports did not clearly define the phenotype or rate of progression of the disease. In some cases, clinical presentation is similar to that described in the childhood onset patients, with progressive limb, facial, extraocular, oropharyngeal and respiratory muscle weakness, but with a slower progression, whereas in other cases, CPEO is the main manifestation [9]. Respiratory insufficiency has been mentioned as a potential cause of death although comprehensive data about the respiratory involvement is not available for all the previously published patients: severe respiratory insufficiency is described in 41% of the reported cases but in the remaining 59% this data is unavailable or superficially described [7, 8, 22].

We identified 16 Spanish and two North American patients, from 13 different families, with TK2 mutations and a late-onset presentation. Exhaustive clinical description is here provided to facilitate earlier and accurate diagnosis and to improve the knowledge of the natural history of this rare, and probably underdiagnosed disorder.

The clinical features and results of the diagnostic tests described in our series show a homogeneous phenotypic pattern in late-onset TK2 deficiency consisting of progressive proximal limb, axial neck flexor and facial muscle weakness frequently associated with ptosis, ophthalmoparesis and bulbar weakness, along with an early and severe, although unrecognized, respiratory involvement. Diaphragmatic weakness is very characteristic, occurring in all of our cases, showing an early onset but slow progression; 12/18 (66.6%) required MV during the evolution of the disease and in 8/18 (44.4%) was the cause for the first medical consultation. This pattern of respiratory involvement was found even in patients who only had an apparently isolated CPEO phenotype. Therefore, it is critical to identify signs of nocturnal hypoventilation during clinical evaluation of these patients, regardless of the severity of the skeletal myopathy. This discrepancy between diaphragmatic and limb weakness was also reflected in some patients with virtually normal 6MWT results, despite using MV (see Table 1). In our series, the capnography was the most sensitive test for detecting the respiratory dysfunction, since it was abnormal even before basal FVC and MIP revealed alterations.

Muscle biopsies showed the typical findings of mitochondrial dysfunction described in most mitochondrial myopathies. However, as in other TK2 deficiency forms, they also revealed dystrophic features which are distinct from the majority of other mitochondrial myopathies. Thus, our data support that the association of both mitochondrial and dystrophic pattern strongly suggest mutations in the *TK2* gene as the underlying cause.

All previously published late-onset patients showed multiple mtDNA deletions, while mtDNA depletion was found only in one of the five cases in whom the mtDNA copy-number was quantified. Our findings corroborate the previous results indicating the presence of multiple mtDNA deletions is more frequent than mtDNA depletion in the late-onset TK2 deficient patients. Previous reports showed that mtDNA depletion is found in the most of early onset patients [7], but our data support that it cannot be considered a valid prognostic marker since it can also be found in late-onset cases.

In muscle MRI the fat muscle replacement was diffuse, resembling many muscular dystrophies and congenital myopathies. Muscle degeneration in MRI was described in five MERRF patients with the m.8344A > G mutation [23], and more recently fatty infiltration has been communicated in patients with single, large-scale deletions of mitochondrial DNA [24]. However, no extensive studies have been published trying to define muscle MRI patterns in different mitochondrial myopathies. So, there is no specific MRI pattern for any mitochondrial myopathy described so far. In our series of TK2 patients although no clear pattern of fat infiltration was detected, we have identified some radiological common features, as the involvement of the sartorius muscle in all cases. This muscle is usually spared until late stages in many genetic muscle diseases (is only affected early in some myofibrillar myopathies, in the Laing distal myopathy and in RYR1-related myopathies (encodes for ryanodine receptor 1 protein) [12, 25–27]), so this finding could be helpful for differential diagnosis.

Serum GDF-15 levels have recently been revealed as a sensitive and specific biomarker for the diagnosis of mitochondrial myopathies [16, 17]. In our series, it proved to be very high in all analysed cases, so it could orientate the molecular diagnosis in a proper clinical context, before the muscle biopsy was performed.

As in other mitochondrial myopathies [19], in our series the cardiopulmonary exercise testing identified a very reduced consumption of oxygen, even in patients with CPEO as a predominant clinical manifestation (P8). This indicates that, although the weakness may not be severe in late-onset TK2 deficiency patients, the exercise capacity is abnormally low, ultimately impairing physical activity.

Noticeably, the p.Lys202del was the most frequent mutation in the *TK2* gene in our series of late-onset patients, which is consistent with the finding that this mutation appears to be restricted to adult-onset cases, since it has not been reported in any infantile-onset patients who have not even harbouring this mutation in a single allele [8]. Nevertheless, it was reported in one patient with childhood-onset, who was compound heterozygous for this mutation and a frameshift mutation, and began showing symptoms at 2.5 years but survived until 8.5 years-old [28]. The eight cases with this mutation in our series were all homozygous supporting the idea that this mutation is associated with a milder effect (age at onset ranging from 25 to 60 years). Interestingly, this mutation has only been identified in 13 unrelated Spanish patients ((11, 13, 26, 27, and this study), 2 related patients from Hispanic ethnic background [10], and one patient from Venezuela (this study) suggesting that it could be a private mutation and that Spanish/Hispanic candidate patients may be amenable for a rapid genetic screening of this mutation. However, haplotype analysis would be required to confirm the possible founder effect of this mutation. The p.Thr108Met mutation was the second most common mutation in this study, however it has been found in infantile and childhood onset cases [6, 7] of different geographic origin.

TK2 deficiency is a severe disorder causing premature death. In recent pre-clinical studies, it has been demonstrated that treatment with pyrimidine nucleosides (dC + dT) in the H126N knock-in mouse model of TK2 deficiency, leads to a prolonged life span in the animals and a restored mtDNA copy number, without significant toxicity [4]. This opens the door to a potential therapeutic intervention in humans with this metabolic hereditary disorder, making it necessary to define sensitive and objective outcomes to assess an eventual response to treatment. Our findings suggest that functional respiratory tests, serum GDF-15 level and the stress cyclometer evaluation are potentially good candidates for monitoring the progression of disease.

## Conclusion

In summary, our study shows that late-onset patients with mitochondrial TK2 deficiency have a consistent and recognizable clinical phenotype, characterized by a progressive myopathy with predominant facial and axial neck flexor weakness, and respiratory involvement, often associated to CPEO. Their prognosis is poor, due to the high risk of early and progressive respiratory insufficiency. Yet, some patients may present with a severe acute respiratory failure. Early detection of respiratory involvement requires an active search in the clinics, even in asymptomatic patients. A small number of rationally designed treatments are being developed for mitochondrial disorders [29], including nucleoside substrate enhancement therapy designed specifically for TK2 deficiency [4]. Therefore, early diagnosis of TK2 deficiency is important as patients could benefit from the existence of a potential therapy.

## Abbreviations

6MWT: 6-min walking test
ATPase: Adenosine triphosphatase
BMI: Body mass index
CK: Creatine kinase
CO_2_: Carbon dioxide
COX: Cytochrome C oxidase
CPEO: Chronic progressive external ophthalmoplegia
dC: Deoxycytidine
dT: Thymidine
FVC: Forced vital capacity
GDF-15: Growth differentiation factor 15
H&E: Hematoxylin and eosin
MIP: Maximal inspiratory pressure
MRC: Muscle Research Council
MRI: Magnetic resonance imaging
mtDNA: Mitochondrial DNA
MV: Mechanical ventilation
MVS: Mercury visual scale
NADH: Nicotinamide adenine dehydrogenase
NGS: Next generation sequencing
PCR: Polymerase chain reaction
SDH: Succinate dehydrogenase
STIR: Shor tau inversion recovery
VO_2_peak: Peak oxygen uptake
